# Brain stem evoked response audiometry of former drug users

**DOI:** 10.5935/1808-8694.20120014

**Published:** 2015-11-20

**Authors:** Tainara Milbradt Weich, Tania Maria Tochetto, Lilian Seligman

**Affiliations:** a MSc. in Human Communication Disorders (Speech and Hearing Therapist); b PhD in Human Communication Disorders (Adjunct Professor in the Speech and Hearing Therapy Department at the Federal University of Santa Maria); c PhD in Industrial Engineering (Adjunct Professor in the Speech and Hearing Therapy Department at the Federal University of Santa Maria)

**Keywords:** hearing, Cannabis, crack, cocaine, street drugs, evoked potentials, auditory, brain stem

## Abstract

Illicit drugs are known for their deleterious effects upon the central nervous system and more specifically for how they adversely affect hearing.

**Objective:**

This study aims to analyze and compare the hearing complaints and the results of brainstem evoked response audiometry (BERA) of former drug user support group goers.

**Methods:**

This is a cross-sectional non-experimental descriptive quantitative study. The sample consisted of 17 subjects divided by their preferred drug of use. Ten individuals were placed in the marijuana group (G1) and seven in the *crack/cocaine* group (G2). The subjects were further divided based on how long they had been using drugs: 1 to 5 years, 6 to 10 years, and over 15 years. They were interviewed, and assessed by pure tone audiometry, acoustic impedance tests, and BERA.

**Results:**

No statistically significant differences were found between G1 and G2 or time of drug use in absolute latencies and interpeak intervals. However, only five of the 17 individuals had BERA results with adequate results for their ages.

**Conclusion:**

Marijuana and crack/cocaine may cause diffuse disorders in the brainstem and compromise the transmission of auditory stimuli regardless of how long these substances are used for.

## INTRODUCTION

Illicit drug use introduces significant disorders in the human body, more specifically in the central nervous system (CNS). Cannabis, cocaine, and *crack* are among the most used drugs.

Tetrahydrocannabinol (THC) is the active chemical in marijuana, a byproduct of plant species *Cannabis sativa.* The hallucinogenic effect in cannabis varies depending on the THC levels found in the plant^1^. *Crack* and cocaine derive from plant species *Erythroxylon coca* and are chemically identical. However, their preparation methods are different^2^. Cocaine is an alkaloid in the form of a water-soluble salt and *crack* is made from the dissolution of cocaine chlorhydrate in water mixed with sodium bicarbonate. The effect of *crack* is more devastating than that of cocaine, as the sensations provoked by the use of *crack* last for a few minutes, thus leading it to be used more frequently^1^.

The auditory consequences of drug abuse have been described in case reports and animal model studies^3^, ^4^, ^5^, ^6^, ^7^, ^8^, ^9^. Some authors have recently looked into the effects of *crack* and cocaine on the auditory pathways and upon central auditory processing.

Moeller et al.^10^ reported a statistically significant difference in P300 amplitudes of a group of former cocaine users when compared against a control group, but failed to observe differences in response latencies. P50, N100, and P200 evoked auditory potential amplitudes may also be reduced and absolute latencies increased in cocaine users^11^.

According to brainstem auditory evoked potential (BAEP) testing, drugs did not introduce evident impairments in the brainstem auditory pathway when users of *crack* and multiple drugs were compared to a control group^12^.

Despite the existing papers on the matter, only a few studies have looked into the effects of marijuana and *crack*/cocaine on BAEP. According to the literature, the alterations introduced by cannabis upon the CNS recede after one month of abstinence^13^, ^14^, but the same is not seen in *crack*/cocaine users, as they cause permanent damage to myelin, neuronal degeneration and glial cells, and hamper the release and capture of neurotransmitters^15^, ^16^, ^17^, ^18^.

This paper aimed to analyze and compare the absolute latencies and interpeak intervals of the BAEPs of former drug user support group goers.

## METHOD

This is a cross-sectional non-experimental descriptive quantitative study. Data sets were collected in the audiology ward of a university hospital. The research work was carried out from April to July of 2011, and the study was approved by the institution's Ethics Committee and granted license n° 23081.019003/2010-40.

Patients were submitted to the following assessments: interview, inspection of the meatus, acoustic impedance measurements (AIM), pure tone audiometry (PTA), and brainstem evoked auditory potential (BAEP) testing.

The interview consisted of questions aimed at finding out more about the patients' auditory complaints, their otological and drug abuse history. Meatus examination was performed to rule out existing outer and middle ear disorders.

Acoustic impedance measurements were made to select individuals based on the sampling exclusion criteria. An *Interacoustics* AZ6 middle ear examination device was used to categorize patients into types A, B, C, As, and Ad^19^.

PTA was done using a *Sibelmed* AC50-D audiometer. Air conduction thresholds were analyzed for frequencies of 250, 500, 1000, 2000, 3000, 4000, 6000, and 8000 Hz, while bone conduction thresholds at 500, 1000, 2000, 3000, and 4000 Hz were assessed through the ascending-descending method. The degree of hearing loss was categorized as described by Lloyd & Kaplan (1978)^20^.

BAEPs were analyzed using an *Intelligent Hearing Systems* (IHS) SMART-EP BOX (IHS, Estados Unidos) two-channel device with four disposable electrodes and in-ear headphones. The electrodes were placed as follows: the negative electrodes were fixated on the right and left mastoid, the positive electrode on the frontal area closer to the vertex, and the common electrode on the frontal area. Click stimuli were used at 80 dBNA.

The patients were comfortably seated and asked to close their eyes. Sedation was not used, and they were advised to remain calm. Auditory pathway nerve conduction was deemed normal when absolute latencies of waves I, III, and V and interpeak intervals I-III, III-V, and I-V had values in accordance with the standard for normality described by Esteves et al.^21^ shown in Table 1 and in agreement with the biological calibration done at the audiology service where the tests were performed, as presented in Table 2.

**Table 1:** Mean values and standard deviations (ms) of absolute latencies and interpeak intervals on BAEP tests of adult subjects with normal hearing according to Esteves et al. (2009)^21^.

	Wave I	Wave III	Wave V	Interpeak I-III	Interpeak III-V	Interpeak I-V
Mean	1.69	3.82	5.59	2.13	1.78	3.90
Standard Deviation	0.13	0.16	0.20	0.14	0.18	0.21

**Table 2:** Mean values and standard deviations (ms) of absolute latencies and interpeak intervals on BAEP tests of young adult subjects with normal hearing according to biologic calibration done at a university hospital.

	Wave I	Wave III	Wave V	Interpeak I-III	Interpeak III-V	Interpeak I-V
Mean	1.67	3.86	5.66	2.18	1.81	3.99
Standard Deviation	0.11	0.14	0.18	0.11	0.14	0.18

The individuals enrolled in this study were recruited from Psychosocial Care Centers and support groups for former alcohol and drug users managed by institution *Amor Exigente.*

Thirty-two subjects of both genders aged between 15 and 35 years of age agreed to join the study. Eighteen males showed up for the interview. All individuals, their parents and guardians read and signed an informed consent term.

The study included individuals without causal factors for hearing loss such as exposure to occupational noise, family history of hearing impairment, and use of ototoxic medication. Subjects above the age of 35, with air-bone gap on PTA, and without type A tympanogram tracings were excluded. A subject presenting type B tympanogram tracings in both ears was excluded.

The individuals found to have disorders on any of the tests applied were referred for further ENT assessment.

The studied sample comprised 17 individuals divided into two groups based on the drug preferentially used:

• Group 1 (G1): 10 former cannabis users.
• Group 2 (G2): 7 former *crack/cocaine* users.

Groups G1 and G2 were divided based on how long the individuals took drugs for: one to five years; six to 10 years; 11 to 15 years; and more than 15 years.

Four members of the cannabis group also tried *crack* or cocaine on occasion, but in considerably lesser amounts and length of time than G2 members. *Crack* and cocaine users were grouped together given the chemical similarity of both drugs.

The collected data sets were arranged and analyzed using descriptive statistics and non-parametric Chi-square and Mann-Whitney tests. Statistical significance was set at 5% (*p ≤* 0.05).

## RESULTS

Groups G1 and G2 were analyzed based on how long their members used drugs: one to five years; six to 10 years; 11 to 15 years; and more than 15 years (Table 3). The subgroup comprising individuals who took drugs for 11 to 15 years was excluded, as none of the enrolled subjects fitted this description.

**Table 3:** Distribution of the study sample per time and type of drug used.

	Cannabis (n = subjects)	*Crack/cocaine* (n = subjects)	Total (n = subjects)
1-5 years	4	3	7
6-10 years	4	4	8
> 15 years	2	0	2
Total	10	7	17

Table 4 shows the complaints mentioned by the subjects categorized by the time for which they took drugs. No statistically significant differences were found between the groups (*p* = 0.615). The most common complaint was difficulty understanding what was said to them in noisy environments, as reported by 12 of the 17 individuals in the sample.

**Table 4:** Auditory complaints reported by former drug users based on time of use and drug type.

	Hearing loss	Tinnitus	Dizziness	Difficulty comprehending speech in noisy environments
1-5 years	3	3	4	4
6-10 years	2	2	2	7
> 15 years	2	1	0	2

Waves I, III and V could not be analyzed for two individuals in G2 because they were absent or poor reproducibility. These results were not included in the analyses of absolute latencies and interpeak intervals.

All other individuals presented waves I, III, and V at 80 dBNA. Five subjects (29%) had normal bilateral response in all absolute latencies and interpeak intervals. No alterations were observed on wave morphology.

Statistically significant differences were not found for absolute latencies and interpeak intervals when right and left ears were compared. Thus, all ears were grouped together. G1 and G2 subjects in the one to five and six to 10 years of drug use subgroups were compared, and no statistically significant differences were found (Mann-Whitney test).

Mean absolute latencies and interpeak intervals for groups G1 and G2 and their subgroups are shown on Table 5.

**Table 5:** Comparison between BAEP mean absolute latencies and interpeak intervals for G1 and G2 for time of drug use.

	1-5 years (ms)		6-10 years (ms)		> 15 years (ms)
	G1 (n = 3)	G2 (n= 2)	G1 (n = 4)	G2 (n = 4)	G1 (n = 1)
I	1.73	1.72	1.62	1.69	1.63
III	3.96	3.93	3.87	3.89	3.74
V	5.82	5.62	5.66	5.79	5.50
I-III	2.23	2.21	2.25	2.20	2.12
III-V	1.86	1.69	1.79	1.90	1.77
I-V	4.09	3.90	4.04	4.10	3.88

Subjects in G2 who took drugs for one to five years when compared to individuals who used drugs for six to 10 years had increased latencies on waves I and III and reduced latencies on wave V. These results may be related to the presence of hearing loss on PTA seen in all G2 individuals who took drugs for one to five years.

Table 6 shows the number of ears with altered findings in interpeak intervals and the possible site of injury: lower brainstem (I-III); upper brainstem (III-V); diffuse alteration (I-V).

**Table 6:** Site of brainstem alteration.

	Ears
Lower brainstem	3
Upper brainstem	1
Diffuse	7

Presence of auditory complaints and BAEP test results were statistically correlated (*p* = 0.0273). This correlation is described on Table 7.

**Table 7:** Correlation between auditory complaints and outcome of brainstem auditory evoked potential testing.

Complaint	Unaltered BAEP (n = subjects)	Altered BAEP (n = subjects)
Hearing loss	1	6
Tinnitus	1	5
Dizziness	2	4
Difficulty comprehending speech in noisy environments	3	9
No complaints	1	0

According to PTA, five individuals had hearing impairment. Two had mild right ear sensorineural hearing loss, one had moderate to severe right ear and moderate left ear sensorineural hearing loss, and two had normal pure tone averages of three frequencies, but had hearing loss starting at 2000 Hz (one unilateral and the other bilateral).

The correlation between PTA and BAEP test results was statistically significant (Table 8). The seven ears with altered PTA had altered BAEP, and the three ears in which waves I, III and V could not be visualized were suggestive of retrocochlear impairment.

**Table 8:** Comparison between pure tone audiometry and brainstem auditory evoked potential tests.

	Normal BAEP	Altered BAEP
	(n = ears)	(n = ears)
Normal PTA	16	11
Altered PTA	0	7
	*p* = 0.0051	

## DISCUSSION

Time of drug use did not affect the occurrence of auditory complaints. The most common complaint was difficulty comprehending speech in noisy environments, followed by hearing loss, dizziness, and tinnitus (Table 4). Another study indicated that *crack* and multiple drug users also complained of tinnitus and balance disorders^12^.

Speech comprehension relies on the anatomic and functional integrity of the auditory system. Communication generally occurs in noisy environments, and individuals - even the ones with normal hearing - end up missing auditory cues^22^ and having a harder time understanding what they are being told^23^.

The use of ototoxic medication may also introduce alterations in the vestibular system and commonly seen adverse effects such as dizziness, accompanied or not by hearing loss and tinnitus^24^.

Although tinnitus is not always connected to auditory pathway dysfunction, at times it is the first symptom of audiological alterations that can only be identified after the establishment of hearing loss, such as hearing loss induced by high sound pressure levels and ototoxicity^25^.

No statistically significant differences were observed in the absolute latencies or interpeak intervals between G1 and G2, regardless of time of drug use. Our findings are in agreement with a study in which the BAEP responses of controls with normal hearing were compared to those of subjects who had taken *crack* for less and more than five years. However, these authors found statistically significant differences in wave V latencies^12^. It should be mentioned that the agreement between these two studies occurred despite the differences in method, as this study enrolled individuals with hearing loss.

Albeit not statistically significant, subjects in G2 who had taken drugs for one to five years had lower wave V absolute latencies than the individuals who had taken drugs for six to 10 years (Table 5). This is contrary to what other authors have observed, in that wave V latencies were lower in individuals who took *crack* and multiple drugs for over five years^12^. Nonetheless, the results seen in our study may be related to the fact that G2 individuals who took drugs for one to five years presented altered PTA and, if the origin of hearing loss in these cases is cochlear, wave V latencies are expected to be lower because of the effect of recruitment^26^.

The impossibility of analyzing waves I, III, and V for three individuals was not reported in other papers on drug users^8^^,^^9^^,^^12^.

Differently from another paper written on *crack* and multiple drug users in which all individuals had normal BAEP^12^, only five subjects had normal absolute latencies and interpeak intervals based on the standards used in this study. The difference in the responses seen in these studies may be related to the fact that the study mentioned above did not enroll drug users with hearing loss in PTA.

However, in our study even the subjects with normal hearing had increased absolute latencies and interpeak intervals, indicating that in this population the use of marijuana, *crack*, or cocaine could impair the transmission of stimuli in the auditory pathways at the level of the brainstem.

In terms of the possible site of dysfunction, the interpeak interval with the most significant increase in latency was I-V, thus indicating that drug use possibly affects the entire brainstem (Table 6).

A study done on the hearing of neonates exposed to cocaine during pregnancy also showed altered stimuli transmission times in the brainstem made evident by the increased interpeak interval latencies and reduced wave I latencies. The authors noted that the differences were subtle, but that the observed responses could suggest subclinical auditory pathway injury^27^. Altered auditory information processing secondary to exposure to cocaine was also described in a study done on animal models^28^.

The effects of cocaine upon medium and long latency evoked auditory potentials has also been investigated. The authors found significant reductions on amplitudes and latencies on P50, N100, and P200^11^ and on amplitudes on P300^10^ suggestive of subcortical and cortical auditory pathway disorder as a consequence of drug use.

Auditory complaints and altered BAEP were statistically correlated (Table 7). Studies done with normal hearing individuals also found a correlation between complaints of dizziness and altered BAEP^29^^,^^30^ and between complaints of tinnitus and increased absolute latencies and III-V and I-V interpeak intervals^31^.

In our study, nine of the 12 individuals who had difficulty comprehending speech in noisy environments had altered BAEP. The group with this complaint in a study that performed electrophysiological hearing tests in individuals without history of drug use had normal test results^32^.

All ears with hearing loss on PTA had altered BAEP. The absence of all waves seen in three ears with mild to moderate hearing loss indicates that these alterations derive from retrocochlear disease^26^. Eleven ears with normal PTA test results had increased absolute latencies and interpeak intervals on BAEP testing (Table 8).

No statistically significant differences were found between the two groups. The results reported in this study suggest that the use of cannabis or *crack/cocaine* may introduce alterations to the auditory pathway on the brainstem - absence of waves I, III, and V; or only auditory information transmission dysfunction - increased absolute latencies and interpeak intervals.

The increase in response times, mainly on interval I-V, implies that these disorders are diffuse, i.e., they affect the entire brainstem.

## CONCLUSION

Altered absolute latencies and interpeak intervals were observed regardless of how long the subjects in this study took drugs for, implying that the use of cannabis and *crack*/cocaine may introduce diffuse brainstem disorders and compromise the transmission of auditory stimuli.
